# Impact of Income and Industry on New-Onset Diabetes among Employees: A Retrospective Cohort Study

**DOI:** 10.3390/ijerph19031090

**Published:** 2022-01-19

**Authors:** Reiko Ishihara, Akira Babazono, Ning Liu, Reiko Yamao

**Affiliations:** 1 Department of Healthcare Management, College of Healthcare Management, Miyama 835-0018, Japan; 2 Department of Health Care Administration and Management, Graduate School of Medical Sciences, Kyushu University, Fukuoka 812-8582, Japan; babazono@hcam.med.kyushu-u.ac.jp (A.B.); 3MD17061M@s.kyushu-u.ac.jp (R.Y.); 3 Department of Preventive Medicine and Community Health, University of Occupational and Environmental Health, Kitakyushu 807-8555, Japan; ning-liu8@med.uoeh-u.ac.jp

**Keywords:** new-onset diabetes, employees, industry, income, socioeconomic status

## Abstract

The purpose of this study was to investigate the impact of income and industry type on the risk of developing diabetes among Japanese workers, including how this impact is affected by sex. A total of 24,516 employees at small- and medium-sized enterprises in Japan aged 40–74 years who underwent health examinations in fiscal years 2010–2015 were included in this retrospective cohort study. Generalized linear regression models were used to assess the association between new-onset diabetes and income and industry. In men, the cumulative incidence rate was significantly higher in the low-income group; it was highest in the transportation and postal service industries. Although income and industry were independent risk factors for developing diabetes in men, an interaction was found between income and industry, which was affected by participants’ sex: in specific industries (i.e., lifestyle-related, personal services, and entertainment services), men had a significantly higher risk of developing diabetes in the high-income group, and women had a significantly higher risk of developing diabetes in the low-income group. These findings highlight important factors to consider in assessing diabetes risk and suggest that efficient primary and secondary prevention should be encouraged in industries where workers have a high risk of diabetes.

## 1. Introduction

Factors such as obesity, lack of physical activity, unhealthy dietary patterns, and smoking are risk factors for diabetes [1,2]. These are known to be influenced by socioeconomic factors such as income, occupation, and education [3,4,5]. Many reports of relationships between socioeconomic factors and the prevalence or incidence of type 2 diabetes by occupational class [6,7,8,9], income level [7,8,10] and educational background exist [6,7,8,9,10].

Variables such as education, occupation, and income, which are commonly used to measure socioeconomic status (SES), reflect a particular exposure [8]. Educational background partially determines adult occupation and income and reflects SES in childhood and adolescence. Occupations reflect specific environmental exposures. Although income may be limited by health status, it reflects educational background and occupation and directly influences the amount of available economic resources; that is, a low income limits particular services and behaviors, such as obtaining necessary care for diabetes prevention and maintaining a balanced diet. Such restriction on access leads to mental stress and directly affects not only health behavior, but also stress hormones, which influence blood glucose levels and insulin tolerance [11].

Diabetes has adverse effects on work capacity, including increased sickness absence and early retirement [12,13]. As workers spend a lot of time in the workplace, the working environment affects their health and well-being [14,15]. As a result, the workplace is a potentially important and underutilized site of prevention. Interventions in the workplace have shown promising results [16,17,18], and the first step in implementing such an approach is to identify industries at high risk for diabetes. Furthermore, in Japan’s social insurance system, where the insurance coverage is determined on a per-workplace basis, identifying industries and workplaces with a high risk of diabetes and implementing efficient primary and secondary prevention programs will contribute to curbing healthcare expenditures [19].

However, research has focused on occupation and occupational class as a variable in investigating the relationship between SES and diabetes, with few studies that estimate the difference in the onset of diabetes depending on the type of industry [20,21,22]. A study of Swedish workers regarding the risk of developing type 2 diabetes reported a large variation across occupational groups; manufacturing workers, professional drivers, and cleaners had 2–3 times higher rates of contracting the disease as compared to university teachers and physiotherapists [20]. In addition, a study in Australia investigated the relationship between the prevalence of diabetes and lifestyle, as well as the biomedical risk factors of diabetes in occupational and industrial groups. They reported that the prevalence rate was highest in the transportation industry, and the risk was most strongly associated with smoking among blue-collar or non-managerial white-collar workers [22]. It is thus evident that research has focused on lifestyle-related factors in addition to occupation and occupational class; thus, other socioeconomic factors are not considered. The risk of diabetes may differ depending on occupational class and job description, even within the same industry; therefore, there may be an interaction between industry and income that reflects the financial resource situation of an individual including occupational class and job description [23].

Sex differences may also affect the association between socioeconomic factors and diabetes. Previous studies have reported that women are more affected by SES than men in the association between diabetes risk and SES [7,8,10,24,25,26]. In a study by Rathmann et al. [25] in Germany, diabetes was associated with low SES, defined by occupational class or income, only in women. In a cross-sectional study by Jongnam et al. [27] in South Korea, an association between diabetes and low income was found only in women. Therefore, as sex differences may affect the relationship between SES and diabetes, it is necessary to consider these when examining this relationship.

As individuals cannot easily change their occupation or income [28], identifying industries and individuals at high risk of diabetes may help implement efficient primary and secondary preventions. Therefore, the purpose of this study was to clarify the effects of income and industry on the risk of developing diabetes among Japanese employees of small- and medium-sized enterprises (SMEs), including how this impact is affected by gender.

## 2. Materials and Methods

### 2.1. Data Sources

We used data on employees at the Fukuoka Branch of the Japan Health Insurance Association, which is an insurer for employees of SMEs and their family members who are younger than 75 years of age. These beneficiaries represent 29.3% of the entire Japanese population [29]. The Fukuoka Branch had 18,377.66 beneficiaries at the end of the 2015 fiscal year, including 10,366.36 employees.

Insurers are required by the Act on Assurance of Medical Care for Elderly People to conduct health examinations focusing on metabolic syndrome for insured persons aged 40–74 and their families. The act implemented measures for promoting the optimization of medical expenses and performing health check-ups. Furthermore, the government requires all health insurance companies to analyze data such as health insurance claims and to develop, announce, implement, and evaluate a project called “Data Health Plan” to maintain and improve the health conditions of subscribers based on the analysis. In this study, we analyzed the health check-ups and claims data linked at the individual subject level. The health check-up data included the dates of check-ups, body mass index (BMI) values, and the results of the blood chemical analysis. Furthermore, a self-reported questionnaire that included participants’ responses to several questions on medical history, comorbidities, and lifestyle factors was also included [30]. The claims data included information on the dates of consultations and treatments, as well as participants’ gender, age, diagnoses, specific treatments, and healthcare expenditure. Furthermore, the health check-up and claims data were linked at the individual subject level for analysis.

### 2.2. Study Population

**Figure 1:**
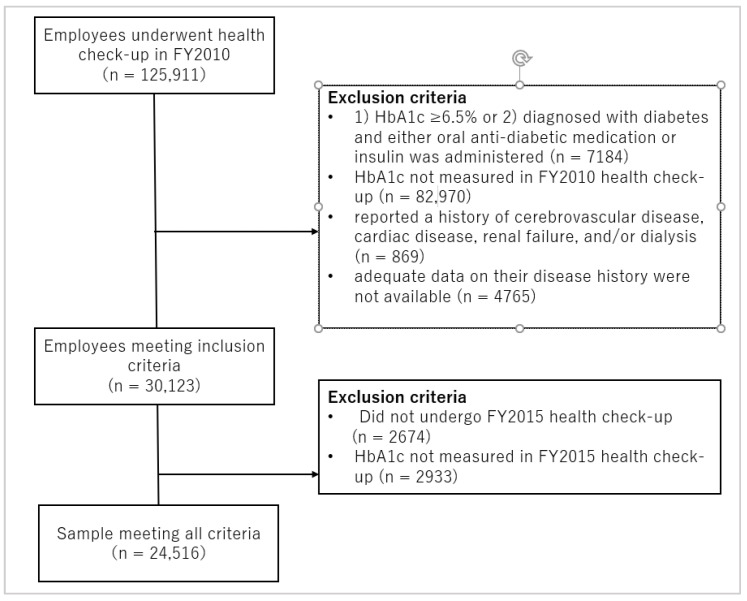
Flowchart of inclusion and exclusion criteria.

Figure 1 shows the inclusion and exclusion criteria, and the participant selection flowchart. We selected non-diabetic individuals from a group of 125,911 employees who had undergone a specific health check-up at the Fukuoka branch of the Japan Health Insurance Association in 2010 as the study’s participants. Individuals were excluded from the study if (1) they reported a history of cerebrovascular disease, cardiac disease, renal failure, and/or dialysis or (2) adequate data on their disease history were not available. Among the 30,123 employees who met the inclusion criteria, 24,516 employees who had undergone a follow up health check-up in 2015 and had had their hemoglobin A1c (HbA1c) values measured were included in the analysis.

### 2.3. Definition of Diabetes

Since the Japan Diabetes Society unit for HbA1c measurement was used in specific health check-ups until 2011, we converted the HbA1c (Japan Diabetes Society) value measured in 2011 to the National Glycohemoglobin Standardization Program unit of measurement [31]. Participants were considered diabetic if (1) their HbA1c values from the check-up exceeded 6.5%, or (2) they were diagnosed with diabetes (International Classification of Diseases 10th revision codes: E10–14) according to the health insurance claim, and either oral anti-diabetic medication or insulin was administered to them.

### 2.4. Categorizations of Variables

The onset of diabetes was defined as the primary outcome. We used three age groups, four income groups, BMI, comorbidities, and smoking status as covariates. Age was categorized as 40–49, 50–59, and ≥60 years based on their age on 1 April 2010. Income was categorized using quartiles of standard monthly income available in the participants’ 2010 health insurance records into those with Q1 (low), standard monthly income <2000 USD; Q2 (lower middle), standard monthly income = USD 2000–2999; Q3 (upper middle), standard monthly income = USD 3000–3799; and Q4 (high), standard monthly income USD ≥ 3800 (USD 1 = 100 Japanese Yen). Comorbidities were noted if participants reported taking medications for hypertension and/or dyslipidemia in the questionnaire. As the questionnaire only enquired about the medication status of these two comorbidities, we used only these two comorbidities. BMI was categorized as BMI < 25.0 or ≥ 25.0, calculated as kg/m^2^ [32]. Smoking status was categorized as current smoker or non-smoker based on the answer to this item in the questionnaire. Occupation was categorized into 18 groups according to the Japan Standard Industrial Classification [33].

### 2.5. Statistical Analysis

First, we conducted a chi-square test to compare the onset of diabetes over the past 5 years including age, income, comorbidity, BMI, smoking, and type of industry. The chi-square test also enabled the comparison of outcomes by sex.

Subsequently, to calculate the odds ratio (OR) of the development of diabetes among participants, generalized linear regression models were created with diabetes onset as the dependent variable and income and the types of industry as covariates; 95% confidence interval (CI) values were calculated. The analyses were performed after adjusting for sex, age, comorbidity, BMI, and smoking status. The following were used as reference categories for covariates: females, 40–49 years of age, Q4 (high) standard monthly income USD ≥ 3800, no comorbidities, BMI < 25.0, non-smoker, medical health care, and welfare services occupation. To assess the interaction between income and industry, we performed a subgroup analysis of each income level adjusted for age, comorbidities, BMI, and smoking by sex.

Data were analyzed using Stata 15.0 (Stata Corp LLC, College Station, TX, USA), and statistical significance was set at *p* < 0.05.

### 2.6. Ethical Considerations

We used administrative claim and health check data; hence, patients were not directly involved in this study, and we did not need to obtain informed consent. The study was approved by the Institutional Review Board of Kyushu University (Clinical Bioethics Committee of the Graduate School of Healthcare Sciences, Kyushu University) (Approval No. 2020335).

## 3. Results

### 3.1. Participant Characteristics

Table 1 summarizes the characteristics of the participants categorized according to the onset of diabetes. In this study, there were a total of 24,516 participants: 4.0% of men and 1.7% of women developed diabetes over five years. The five-year cumulative incidence of developing diabetes was significantly higher in men than in women and in the oldest than in the youngest group (*p* < 0.001). A total of 10.4% of men and 50.9% of women were in the low-income quartile. In the high-income quartile, these percentages were 34.5% and 8.1%, respectively. The cumulative incidence of diabetes was significantly higher in men in the low-income group in men (*p* < 0.001) and women (*p* = 0.002), respectively. In addition, the cumulative incidence of diabetes was significantly higher in participants taking medication due to hypertension and dyslipidemia, those with a BMI ≥ 25.0, and those who smoked (*p* < 0.001). By industry, the cumulative incidence was significantly higher in participants working in the transport and postal services industries than in others (4.7%, *p* < 0.001) and significantly lower in the medical, health care, and welfare industries (1.6%, *p* < 0.001). Similar results were obtained by industry for men.

### 3.2. Odds Ratios and 95% Confidence Intervals for the Onset of Diabetes

Table 2 shows the risk of diabetes (OR and 95% CI data), as determined by the generalized linear regression model by sex. In men, univariate analysis demonstrated that the transport and postal service industries (OR: 1.32, 95%CI: 1.08–1.62) as risk factors and the medical, health care, and welfare industries (OR: 0.60, 95% CI: 0.38–0.95), as well as the manufacturing industry (OR: 0.75, 95%CI: 0.62–0.91), as preventive factors were significantly associated with the onset of diabetes. In addition, multivariate analysis with reference to the medical, health care, and welfare industries revealed that the wholesale and retail trade industry (OR: 1.72, 95%CI: 1.06–2.79) and real estate and goods rental and leasing industries (OR: 2.07, 95%CI: 1.06–4.04) were significantly associated with the onset of diabetes. No significant OR was observed in either the univariate or multivariate analysis in women.

Table 3 shows results of the same analysis conducted for income level by type of industry adjusted for age, comorbidities, BMI, and smoking, by sex. In men with a high income level, the living-related, personal services, and entertainment services industry (OR: 4.16, 95%CI: 1.16–14.86); the other services industry (OR: 3.10, 95%CI: 1.29–7.47); and wholesale and retail trade industry (OR: 2.52 95%CI: 1.13–5.65) showed significantly high ORs. In contrast, for women with a low income level, the living-related, personal services, and entertainment services industry (OR: 4.04. 95%CI: 1.34–12.18) showed significantly high ORs.

### 3.3. Summary of Research Results

The cumulative incidence rate was the highest for the transport and postal services and lowest for the medical, health care, and welfare industry. There was a clear difference in the risk of developing diabetes between men and women. Specifically, in men, income and industry were independent risk factors for developing diabetes. Furthermore, there was an interaction between income and industry, where men had a significantly higher risk of developing diabetes in the high-income group compared to women who had a significantly higher risk of developing diabetes in the low-income group.

## 4. Discussion

As most research has focused on lifestyle-related factors related to diabetes, a lack of research investigating the relationship between SES and the risk of developing diabetes by industry type exists. Thus, the present study focused on the risk of developing diabetes by industry in Japan and revealed the following important findings: (1) The cumulative incidence rate of developing diabetes was significantly higher in men than women, with a significantly higher cumulative incidence rate in men in the low-income than high-income group. In addition, differences were found in the cumulative incidence rate between industries in men, with the transport and postal services being the highest and medical, health care, and welfare the lowest. In contrast, (2) multivariate analysis showed a clear difference in the risk of developing diabetes between men and women, and income and industry were independent risk factors for developing diabetes in men. In addition, an interaction between income and industry was found, and in specific industries (i.e., living-related, personal services, and entertainment services industries), the income groups with higher risk of developing diabetes by industry were opposite for men and women; that is, even in the same industry, men had a significantly higher risk of developing diabetes in the high-income group, whereas women had a significantly higher risk of developing diabetes in the low-income group. Each finding is discussed in detail below.

First, the 5-year cumulative incidence of diabetes among Japanese SME employees was 4.0% for men and 1.7% for women, about twice as high for men than women. This was quite low compared to 16.1% for men and 8.8% for women, which was the percentage of people who had been referred to as having “diabetes” at medical institutions and medical examinations in the 2010 National Health and Nutrition Survey [34]. While the proportion of elderly people was high in this survey, about half of the subjects in this study were in their 40s, mainly in the younger generation. However, compared to 8.0% for men and 3.4% for women found in the results of the National Health and Nutrition Survey [34], the cumulative incidence rate in this study was low. This may be because the subjects of this study were only workers; thus, worker effects may exist.

Furthermore, in men, the cumulative incidence rate of diabetes was significantly higher in the low-income group, with transport and postal services having the highest rate at 5.0%, and medical, health care, and welfare industries the lowest rate at 2.5%. The Gallup survey of 90,000 American workers reported that the prevalence of diabetes was highest in the transport industry at 10.3% and lowest at 5.1% for physicians [22]. In this study, transport workers had a high rate of obesity and low physical activity. This is in line with research that reports that obesity, smoking, and alcohol intake are high in high-risk industries [22]. Similarly, in a Swedish report [20], the prevalence of diabetes in vehicle drivers was the highest at 7%, and the proportion of obese and smoking individuals was also high. In this study, the risk of developing diabetes in men in the transport and postal services showed a significant OR in univariate analysis, but no significant difference was found after adjusting for age, income, comorbidity, BMI, and smoking status. This suggests that the risk of developing diabetes in men in the transport and postal services industry is explained by BMI, smoking, and income. In addition, long sitting times [35], irregular working hours [36], and long working hours [37] increase the risk of developing diabetes, which is prevalent in these industries.

The cumulative incidence rate was the lowest in the medical, health care, and welfare industries, which was similar to that reported in the United States [22]. This may be due to the high health literacy present in these industries. Health literacy is defined as the cognitive and social skills that determine the motivation and ability of individuals to gain access to, understand, and use information in ways that promote and maintain good health [38]. There are reports on the association between health literacy and a healthy lifestyle [39,40], including an inverse correlation between health literacy and the prevalence of metabolic syndrome in Japanese men [41]. Medical professionals, including physicians, are in a position to provide health information to patients and local residents and guide them in engaging in healthy behavior. Therefore, high health literacy, such as taking the initiative to engage in healthy behavior, leads to a low risk of developing diabetes.

Second, multivariate analysis revealed a clear difference between men and women in the risk of developing diabetes. Specifically, income and industry were independent risk factors for developing diabetes in men; that is, the wholesale and retail trade, as well as the real estate and goods rental and leasing industry, was an independent risk factor for developing diabetes, even after adjusting for age, income, BMI, smoking, and comorbidities. This is not surprising, as it has been shown that lifestyle-based factors, such as obesity, smoking, unhealthy diet, and lack of physical activity [1], as well as shift work [36], long sitting times [35], and psychological stress [42], may promote diabetes. Therefore, the finding that men working in the wholesale and retail trade industry, as well as the real estate and goods rental and leasing industry, are at higher risk for developing diabetes may be explained by industry-specific diabetes risk factors, such as psychological stress in their workplace [42].

Many studies have reported that socioeconomic factors, including income, are associated with the onset of diabetes only in women; however, no association in women was found in this study. In a study by Rathmann et al. [25] in Germany, diabetes was associated with low SES, defined by occupational class or income, only in women. This study targeted a general population aged 55–74 years, and those without regular employment, such as housewives, used their spouse’s occupation as a proxy or used household income. In a cross-sectional study of the general population aged >30 years by Jongnam et al. [27] in South Korea, the association between diabetes and low income was found only in women using their annual household income. Therefore, many of these studies targeted the general population and used their annual household income as the income variable. However, this study targeted workers and used individual monthly income as the income variable. In Japan, the proportion of female non-employees, non-regular employees, and part-time workers is high, and the proportion of low-income workers is high compared to men [37]; a large difference between individual and household income is present in women. Consequently, this may explain how no association between income and the incidence and risk of diabetes in women was observed in this study.

Finally, there was an interaction between income and industry. Interactions were observed between specific industries and high-income groups for men and between specific industries and low-income groups for women. This may be due to potential risk factors for diabetes in these industries, and different income levels between men and women may reflect different work contents and environments. In addition, this finding within the same industry suggests that the mechanism by which income affects the onset of diabetes is different between men and women. There are some reports regarding the relationship between income and diabetes risk, stating that high-income countries have a high risk of developing diabetes in the low-income group [7,25,43]; conversely, in countries that have experienced rapid economic growth during the previous decade, such as China [44] and Thailand [45], the risk is high in the high-income group. In a Chinese study [44], a positive correlation was found between income and education with the onset of diabetes, and this result was reported to be influenced by obesity. Similarly, in the men in this study, the results were consistent with those of countries with rapid economic growth in the living-related, personal services, and entertainment services industries; service industry; and the wholesale and retail trade industries, in which interactions were observed. In other words, people with high incomes in these types of industries may have increased risk of diabetes due to obesity. Long working hours and shift work, which are unique to the above industries [46,47], cause circadian misalignment and increase the risk of obesity through the disruption of metabolic processes without adequate sleep duration and quality [48,49]. In addition, shift work may interfere with choosing healthy food options and promote overeating or the consumption of nutrient-poor and energy-dense foods during and following work [50,51]. In response to these situations, obesity prevention measures should be implemented in the workplace, such as ensuring that employees have time to eat and exercise during work hours, and adjusting shift schedules such that they have adequate time to sleep.

In contrast, for women in this study, the risk of developing diabetes was (1) higher among the low-income group in the living-related, personal services, and entertainment services industries and (2) consistent with reports from high-income countries. Furthermore, low-income limits access to services and resources, such as necessary care for diabetes prevention and a balanced diet [11]. Previous studies have shown that women are more strongly affected by lower incomes than men in the association between diabetes risk and income [7,8,10,24,25]. This may be due not only to the abovementioned physical restrictions, but also due to women being more burdened with work, housework, and childcare than men; thus, women are more time-constrained and mentally burdened.

Although several important insights were gained from this study, several limitations were also present that should be considered. First, we did not consider the changes in income over time since only the data obtained in 2010 were used to categorize participants into income groups. However, this likely introduced little to no bias, since changing jobs is uncommon in the Japanese population [52]. Second, we used the standardized monthly income of individuals as the income levels in this study. As the insurance association had only data on standardized monthly income, we were not able to define household size or other assets. Future research should consider including this information to obtain more precise results. Third, this study did not consider socioeconomic factors other than industry and income, such as occupation, occupational class, and educational background, which are associated with the development of obesity and diabetes [6,7,8,9,10]. As the insurance data did not include this information, they could not be considered in this study. However, given the information obtained from income, some occupational classes and occupational types could be inferred. Fourth, we did not consider factors such as family history that may influence the progression of diabetes. The check-up results and claims data used in this study did not include this information. Future research should consider including the information to obtain more precise results. Finally, a selection bias may have occurred because the study was not a randomized controlled study; that is, participants included those who regularly underwent medical check-ups. Therefore, they were more aware and conscious of their health than those who did not undergo regular medical check-ups.

Since workers spend most of their time in the workplace, there is a strong link between the work environment and health. Therefore, the above findings have demonstrated that it is necessary to take an approach from both aspects of improving the working environment, as well as changing workers’ behavior in the disease management of workers. In addition, the government of Japan has strategically promoted health and productivity management as an approach to consider employee health management from the perspective of corporate management [53]. In these efforts, if the prevalence and risk of developing diabetes by industry are clear, employers and insurers can target narrow subjects for diabetes prevention and management programs. Socioeconomic factors such as income cannot be easily changed. More efficient disease management programs that consider the diverse socioeconomic backgrounds of individual workers—for example, a program on improving the lifestyle and working environment of workers and enables them to see a doctor whenever they need—should be implemented. In future research, risk assessment based on both industry and occupation will further clarify the target populations of such programs and enable the implementation of more specific programs.

## 5. Conclusions

This study focused on the risk of developing diabetes by industry in Japan. The cumulative incidence rate was the highest for the transport and postal services and lowest for the medical, health care, and welfare industry. There was a clear difference in the risk of developing diabetes between men and women. Specifically, in men, income and industry were independent risk factors for developing diabetes. Furthermore, there was an interaction between income and industry. For men, the risk of developing diabetes was higher in the high-income groups in the living-related, personal services, and entertainment services industries; service industry; and the wholesale and retail trade industries. Meanwhile, for women, the risk was higher in the lower income groups in the living-related, personal services, and entertainment services industries. This suggests that the mechanism by which income affects the onset of diabetes is clearly different between men and women.

These results suggest that efficient primary and secondary prevention should be especially encouraged in industries that carry a high risk of diabetes. Furthermore, the assessment of diabetes risk through a consideration of individuals’ diverse socioeconomic backgrounds, as well as the industry in which they operate, is also crucial. This study thus provides a starting point for further research on this topic and highlights the relevant factors that influence diabetes risk. To implement efficient diabetes prevention and management programs, high-risk industries and individuals should be targeted by adopting measures based on their working environments and socio-economic backgrounds.

## Figures and Tables

**Table 1:** Participant characteristics.

**Title**	**All**				**Male**				**Female**			
	**DM+**		**Total**	***p*** **Value**	**DM+**		**Total**	***p*** **Value**	**DM+**		**Total**	***p*** **Value**
	***n***	**(%)**	***n***		***n***	**(%)**	***n***		***n***	**(%)**	***n***	
	773	(3.2)	24516		620	(4.0)	15474		153	(1.7)	9042	
**Sex**												
Male	620	(4.0)	15,474	<0.001								
Female	153	(1.7)	9042									
**Age Category**												
40–49	332	(2.6)	12783	<0.001	275	(3.4)	8197	<0.001	57	(1.2)	4586	<0.001
50–59	316	(3.4)	9307		244	(4.4)	5585		72	(1.9)	3722	
60–74	125	(5.2)	2426		101	(6.0)	1692		24	(3.3)	734	
**Income Level (USD) Quartile**												
Q_1_: <2000	187	(3.0)	6219	0.704	87	(5.4)	1615	<0.001	100	(2.2)	4604	0.002
Q_2_: 2000–2999	175	(3.3)	5236		143	(4.8)	2977		32	(1.4)	2259	
Q_3_: 3000–3799	214	(3.1)	6990		201	(3.6)	5540		13	(0.9)	1450	
Q_4_: ≥3800	197	(3.2)	6071		189	(3.5)	5342		8	(1.1)	729	
**Hypertension with Medication**												
Yes	338	(5.6)	6035	<0.001	272	(6.1)	4488	<0.001	66	(4.3)	1547	<0.001
No	435	(2.4)	18481		348	(3.2)	10986		87	(1.2)	7495	
**Dyslipidemia with Medication**												
Yes	448	(4.8)	9328	<0.001	352	(5.4)	6486	<0.001	96	(3.4)	2842	<0.001
No	325	(2.1)	15187		268	(3.0)	8987		57	(0.9)	6200	
**BMI**												
<25	350	(1.9)	18423	<0.001	279	(2.6)	10912	<0.001	71	(0.9)	7511	<0.001
≥25	423	(6.9)	6093		341	(7.5)	4562		82	(5.4)	1531	
**Smoking**												
Yes	377	(4.1)	9257	<0.001	340	(4.5)	7634	0.005	37	(2.3)	1623	0.043
No	395	(2.6)	15,243		279	(3.6)	7830		116	(1.6)	7413	
**Types of Industry**												
Agriculture, forestry, and fisheries	4	(6.3)	64	0.156	2	(6.1)	33	0.547	2	(6.5)	31	0.040
Mining and stone quarrying	2	(3.1)	64	0.990	2	(3.4)	59	0.809	0	(0.0)	5	0.769
Construction	79	(3.8)	2077	0.076	70	(4.1)	1699	0.801	9	(2.4)	378	0.289
Manufacturing	156	(2.9)	5444	0.169	133	(3.3)	4090	0.004	23	(1.7)	1354	0.984
Electricity, gas, heat supply, and water	5	(5.1)	99	0.279	4	(5.4)	74	0.539	1	(4.0)	25	0.370
Information and communications	18	(2.0)	880	0.945	15	(3.4)	446	0.482	3	(2.2)	134	0.621
Transport and postal services	130	(4.7)	2788	<0.001	126	(5.0)	2527	0.006	4	(1.5)	261	0.839
Wholesale and retail trade	167	(3.5)	4793	0.143	123	(4.4)	2775	0.207	44	(2.2)	2018	0.054
Finance and insurance	13	(2.6)	497	0.489	9	(4.3)	211	0.847	4	(1.4)	286	0.696
Real estate and goods rental and leasing	18	(4.0)	454	0.318	17	(5.7)	296	0.124	1	(0.6)	158	0.298
Scientific research, professional and technical services	24	(3.8)	630	0.339	23	(5.0)	463	0.284	1	(0.6)	167	0.269
Accommodations, food and beverage services	13	(3.2)	402	0.926	8	(3.4)	234	0.644	5	(3.0)	168	0.193
Living-related and personal services and entertainment services	17	(3.5)	485	0.654	12	(4.4)	274	0.751	5	(2.4)	211	0.440
Education and learning support	4	(1.9)	216	0.272	4	(2.7)	147	0.425	0	(0.0)	69	0.274
Medical, health care, and welfare	55	(1.6)	3343	<0.001	20	(2.5)	797	0.027	35	(1.4)	2546	0.143
Compound services	0	(0.0)	42	0.242	0	(0.0)	19	0.373	0	(0.0)	23	0.529
Other services	55	(2.8)	1999	0.284	47	(3.8)	1232	0.721	8	(1.0)	767	0.145
Government services	13	(2.4)	539	0.319	5	(5.1)	98	0.579	8	(1.8)	441	0.839

*p*-values were calculated using chi-squared test. DM, diabetes mellitus; BMI, body mass index; OR, odds ratio; CI, confidence interval. USD 1 = 100 Japanese Yen.

**Table 2:** Odds ratios and 95% confidence intervals for the onset of diabetes.

Title	Male (*n* = 15,474)						Female (*n* = 9042)					
	Univariate			Multivariate			Univariate			Multivariate		
	OR	95%CI		OR	95%CI		OR	95%CI		OR	95%CI	
**Age Category**												
50–59	1.32	1.10	1.57	1.26	1.05	1.52	1.57	1.10	2.22	1.11	0.76	1.61
60–74	1.83	1.45	2.31	1.59	1.21	2.09	2.69	1.66	4.36	1.55	0.90	2.65
**Income Level (USD) Quartile**												
Q_1_: <2000	1.55	1.20	2.01	1.31	0.97	1.77	2.00	0.97	4.13	1.53	0.70	3.35
Q_2_: 2000–2999	1.38	1.10	1.72	1.38	1.09	1.75	1.30	0.59	2.82	1.23	0.54	2.79
Q_3_: 3000–3799	1.03	0.84	1.26	1.07	0.87	1.32	0.82	0.34	1.98	0.88	0.35	2.20
**Hypertension with Medication**												
Yes	1.97	1.68	2.32	1.54	1.29	1.82	3.79	2.74	5.25	2.08	1.46	2.95
**Dyslipidemia with Medication**												
Yes	1.87	1.59	2.20	1.59	1.35	1.88	3.77	2.71	5.24	2.60	1.84	3.69
**BMI**												
≥25	3.08	2.62	3.62	2.77	2.34	3.28	5.93	4.29	8.19	4.16	2.95	5.87
**Smoking**												
Yes	1.26	1.07	1.48	1.41	1.19	1.66	1.47	1.01	2.13	1.64	1.11	2.42
**Types of Industry**												
Construction	1.03	0.80	1.33	1.43	0.86	2.38	1.44	0.73	2.85	1.89	0.88	4.04
Manufacturing	0.75	0.62	0.91	1.22	0.76	1.98	1.00	0.64	1.57	0.97	0.55	1.69
Information and communications	0.83	0.49	1.40	1.50	0.75	2.98						
Transport and postal services	1.32	1.08	1.62	1.46	0.89	2.38	0.90	0.33	2.45	0.88	0.30	2.56
Wholesale and retail trade	1.14	0.93	1.39	1.72	1.06	2.79	1.41	0.99	2.01	1.20	0.74	1.95
Finance and insurance	1.07	0.55	2.09	1.80	0.80	4.05	0.82	0.30	2.23	1.27	0.43	3.77
Real estate and goods rental and leasing	1.47	0.90	2.42	2.07	1.06	4.04						
Scientific research, professional and technical services	1.26	0.82	1.93	1.78	0.96	3.29						
Accommodations, food and beverage services	0.85	0.42	1.72	1.38	0.59	3.19						
Living-related and personal services and entertainment services	1.10	0.61	1.97	1.64	0.78	3.45	1.42	0.58	3.51	1.79	0.68	4.74
Medical, health care, and welfare	0.60	0.38	0.95	1.00	(reference)		0.75	0.52	1.10	1.00	(reference)	
Other services	0.95	0.70	1.28	1.35	0.79	2.32	0.59	0.29	1.21	0.63	0.29	1.39
Government services							1.08	0.53	2.21	1.21	0.54	2.71

Only those occupational groups with a total of more than 200 participants by sex are shown. BMI, body mass index; OR, odds ratio; CI, confidence interval. USD 1 = 100 Japanese Yen.

**Table 3:** Odds ratios and 95% confidence intervals for the onset of diabetes for each industry by income level and sex.

**Male (*n* = 15,474)**	**Income Level (USD) Quartile**											
	**Q_1_ (*n* = 1615)**			**Q_2_ (*n* = 2977)**			**Q_3_ (*n* = 5540)**			**Q_4_ (*n* = 5,42)**		
	**OR**	**95%CI**		**OR**	**95%CI**		**OR**	**95%CI**		**OR**	**95%CI**	
**Types of Industry**												
Construction	0.82	0.25	2.67	1.70	0.47	6.15	1.16	0.43	3.09	1.86	0.78	4.44
Manufacturing	0.41	0.13	1.26	1.41	0.42	4.70	0.86	0.33	2.22	2.24	0.99	5.05
Information and communications							1.11	0.33	3.75	2.51	0.86	7.33
Transport and postal services	0.54	0.20	1.45	2.11	0.64	6.99	1.07	0.40	2.82	1.88	0.73	4.86
Wholesale and retail trade	0.91	0.30	2.73	1.88	0.53	6.70	1.35	0.52	3.50	2.52	1.13	5.65
Finance and insurance										2.32	0.76	7.05
Real estate and goods rental and leasing				2.21	0.42	11.56	1.81	0.49	6.62	1.71	0.43	6.76
Scientific research, professional and technical services				2.54	0.58	11.21	1.48	0.45	4.86	2.57	0.96	6.90
Accommodations, food and beverage services							0.75	0.14	3.99	1.51	0.30	7.49
Living-related and personal services and entertainment services							1.74	0.53	5.70	4.16	1.16	14.86
Education and learning support							1.34	0.31	5.86			
Medical, health care, and welfare	1	(reference)		1	(reference)		1	(reference)		1	(reference)	
Other services	0.63	0.20	1.98	1.11	0.29	4.32	0.70	0.23	2.11	3.10	1.29	7.47
**Female (*n* = 9042)**	**Income Level (USD) Quartile**											
	**Q_1_ (*n* = 4604)**			**Q_2_ (*n* = 2259)**			**Q_3_ (*n* = 1450)**			**Q_4_ (*n* = 729)**		
	**OR**	**95%CI**		**OR**	**95%CI**		**OR**	**95%CI**		**OR**	**95%CI**	
**Types of Industry**												
Construction	2.28	0.70	7.43	1.37	0.29	6.46	2.83	0.50	16.18			
Manufacturing	1.29	0.59	2.81	1.36	0.49	3.77						
Transport and postal services	1.04	0.22	4.83	1.17	0.24	5.66						
Wholesale and retail trade	1.76	0.88	3.51	0.77	0.24	2.47	0.46	0.05	4.09	0.96	0.10	9.10
Finance and insurance	1.27	0.15	10.56				1.68	0.19	14.87	0.96	0.18	5.28
Living-related and personal services and entertainment services	4.04	1.34	12.18									
Medical, health care, and welfare	1	(reference)		1	(reference)		1	(reference)		1	(reference)	
Other services	1.21	0.48	3.08									
Government services	1.42	0.51	3.95	2.35	0.49	11.28						

Only those occupational groups with a total of more than 200 participants by sex or more than 50 participants by sex and occupational income are shown. JPY, Japanese yen; OR, odds ratio; CI, confidence interval. Q_1_: ≥USD 2000, Q_2_: USD 2000–2999, Q_3_: USD 3000–3799, Q_4_: ≥USD 3800.

## Data Availability

Data were obtained from the Fukuoka Branch of the Japan Health Insurance Association and are available from the corresponding author with the permission of the Fukuoka Branch of the Japan Health Insurance Association.
